# Supplementary material for: A shared genetic contribution to breast cancer and schizophrenia
Source: Nat Commun. 2020 Sep 15;11:4637. doi: 10.1038/s41467-020-18492-8 (PMC7492262; doi:10.1038/s41467-020-18492-8)
Supplement: Supplementary file 1 — Supplementary Information [file 41467_2020_18492_MOESM1_ESM.pdf]

A Shared Genetic Contribution to Breast Cancer and Schizophrenia

Lu and Song et al.

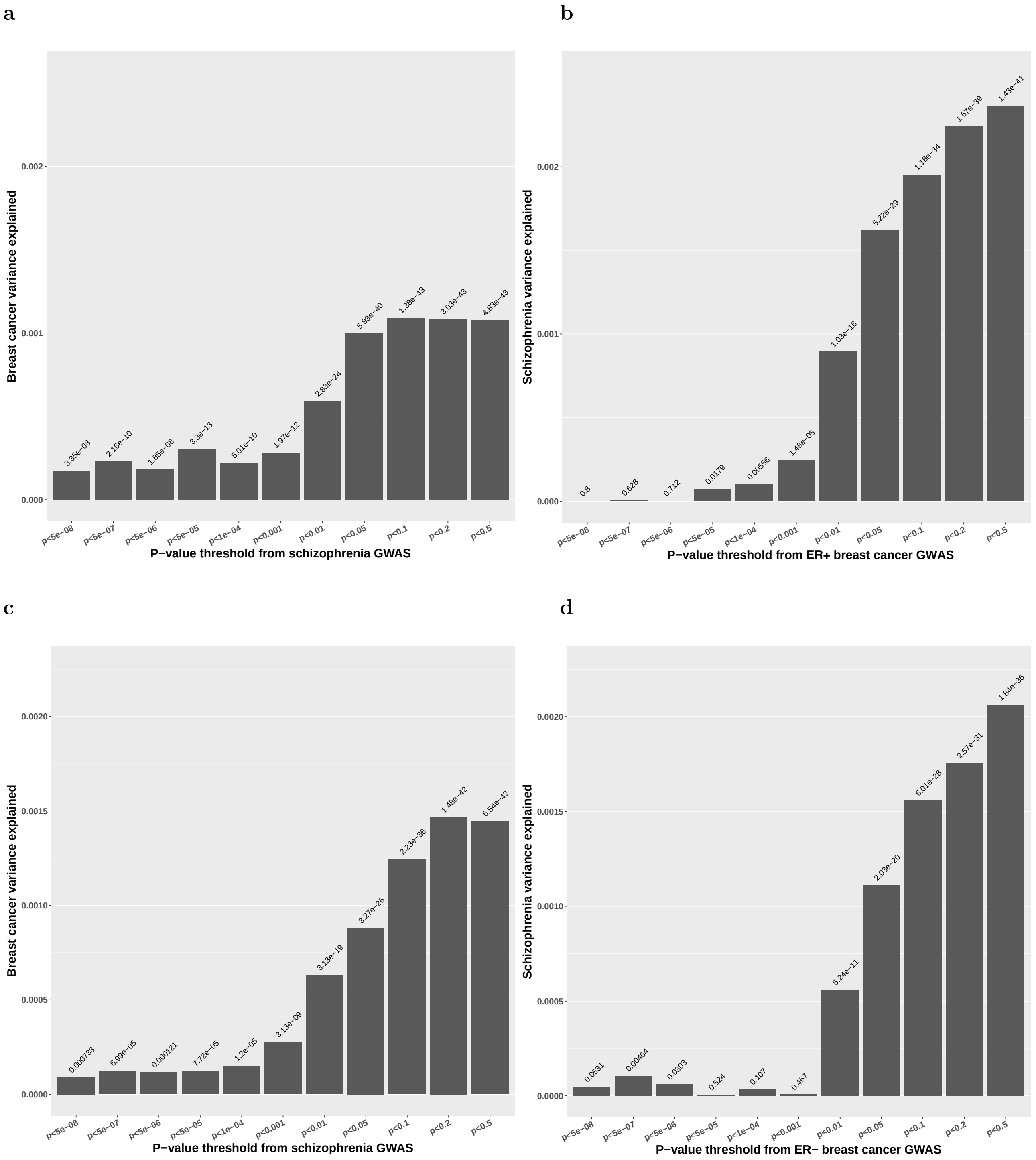

Supplementary Figure 1. Genetic associations between ER-positive/-negative breast cancer and schizophrenia based on GWAS summary statistics of breast cancer and schizophrenia. a, PRS for schizophrenia associated with risk of ER-positive breast cancer. b, PRS for ER-positive breast cancer associated with risk of schizophrenia. c, PRS for schizophrenia associated with risk of ER-negative breast cancer. d, PRS for ER-negative breast cancer associated with risk of schizophrenia. We performed PRS analysis based on GWAS summary statistics. We plotted the variance of one disease ($\boldsymbol{Y}$ axis) explained by the genetic markers associated with the other disease under a $\boldsymbol{P}$-value threshold ($\boldsymbol{X}$ axis). The number above the bar indicates the statistical significance of the genetic association (two-sided $\boldsymbol{P}$-value). ER, estrogen receptor; GWAS, genome-wide association study; PRS, polygenic risk score.

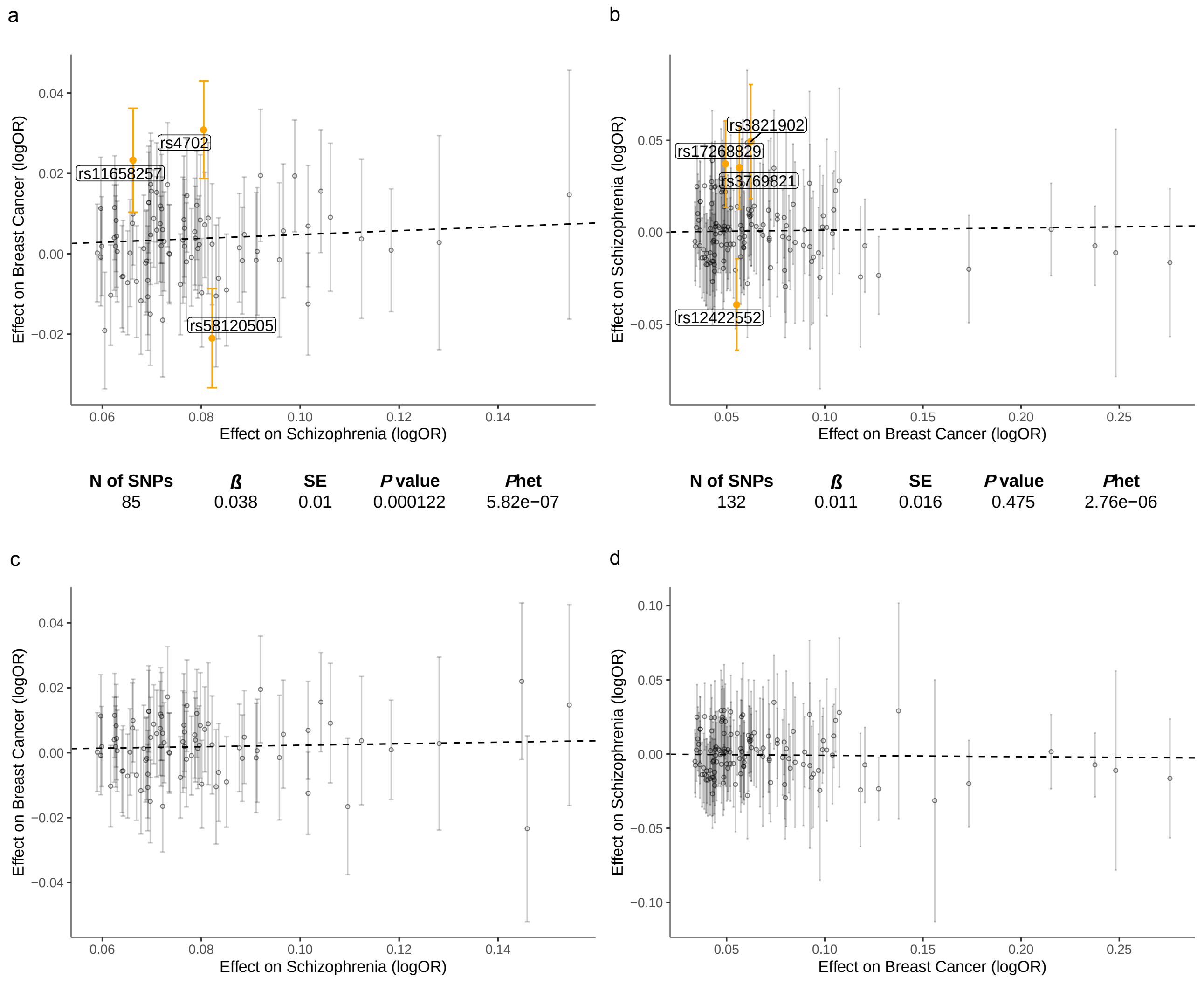

a

N of SNPs	ß	SE	*P* value	*P*het
85	0.038	0.01	0.000122	5.82e−07

b

N of SNPs	ß	SE	*P* value	*P*het
132	0.011	0.016	0.475	2.76e−06

c

N of SNPs	ß	SE	*P* value	*P*het
80	0.023	0.01	0.0236	0.0632

d

N of SNPs	ß	SE	*P* value	*P*het
125	−0.009	0.016	0.589	0.0616

Supplementary Figure 2. Associations of lead SNPs (after filtering for outliers or heterogeneity) for schizophrenia with the risk of breast cancer, and vice versa. a, Lead SNPs for schizophrenia (outliers removed according to Cook's Distance). b, Lead SNPs for breast cancer (outliers removed according to Cook's Distance). c, Lead SNPs for schizophrenia (SNPs iteratively removed until heterogeneity was non-significant). d, Lead SNPs for breast cancer (SNPs iteratively removed until heterogeneity was non-significant). We plotted the associations of genetic markers for one disease (the effect size shown as X axis) with the other disease (Y axis) based on GWAS summary statistics. Data are presented as OR (dots) and 95% confidence interval (error bars). Orange dots denotes variants of P¡0.05 after false discovery rate (FDR) adjustment. We then derived the average effect of the set of genetic markers on the other disease using a global test. The dash line indicates this effect size, while the full statistical results were reported below the plot. P-values are two-sided. N, number; OR, odds ratio; Phet, P for heterogeneity; SD, standard deviation; SE, standard error; SNPs, single nucleotide polymorphisms.

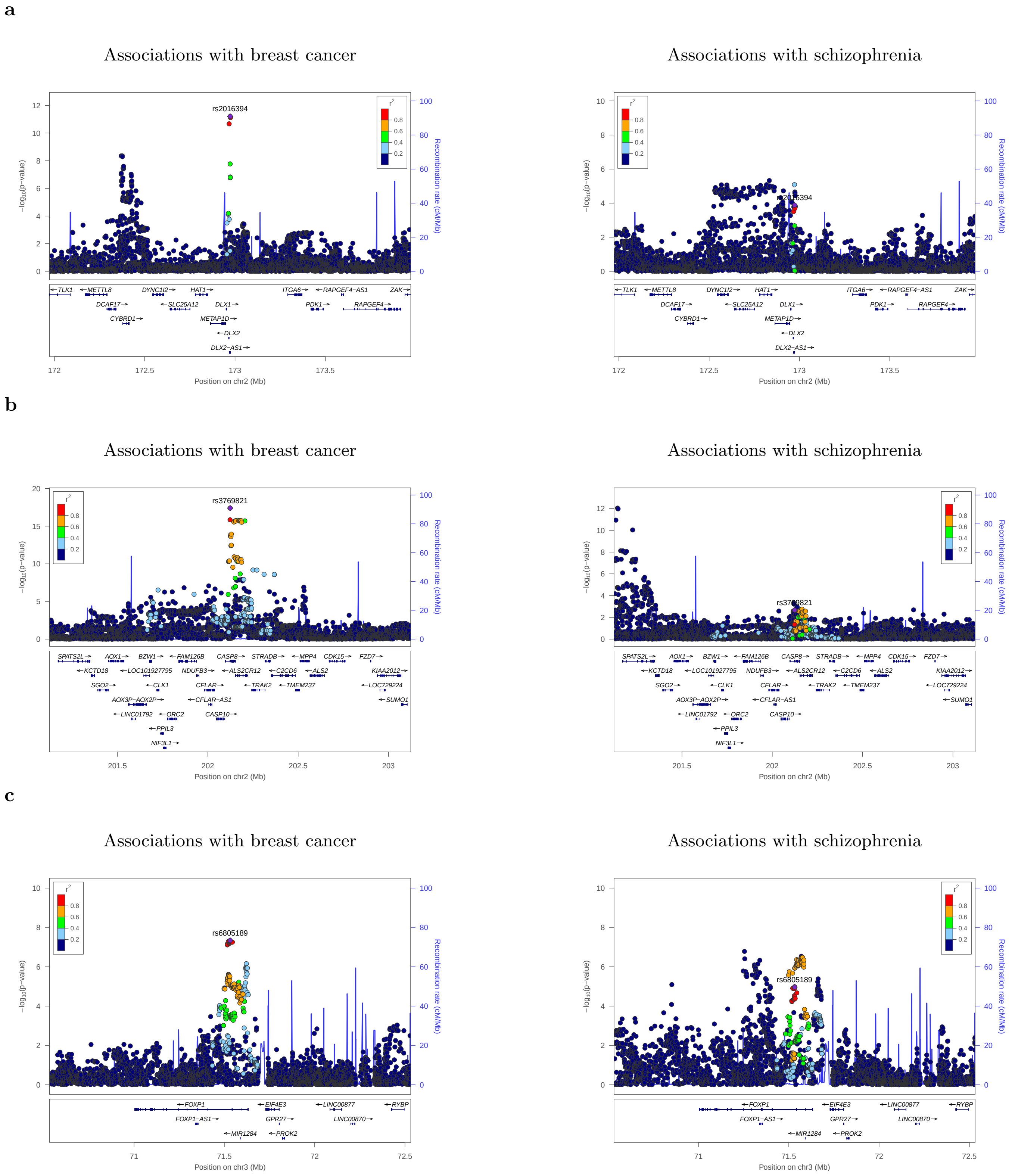
a
Associations with breast cancer
rs2016394
Associations with schizophrenia
rs2016394
Position on chr2 (Mb)
TLK1
METTL8
DCAF17
CYBRD1
DYNC1I2
SLC25A12
HAT1
DLX1
METAP1D
DLX2
DLX2-AS1
ITGA6
PDK1
RAPGEF4-AS1
RAPGEF4
ZAK
b
Associations with breast cancer
rs3769821
Associations with schizophrenia
rs3769821
Position on chr2 (Mb)
SPATS2L
KCTD18
SGO2
AOX1
AOX3P-AOX2P
LINC01792
BZW1
LOC101927795
CLK1
PPIL3
NIF3L1
ORC2
FAM126B
NDUFB3
CFLAR
CFLAR-AS1
CASP10
CASP8
ALS2CR12
STRADB
TRAK2
C2CD6
TMEM237
MPP4
ALS2
CDK15
FZD7
KIAA2012
LOC729224
SUMO1
c
Associations with breast cancer
rs6805189
Associations with schizophrenia
rs6805189
Position on chr3 (Mb)
FOXP1
FOXP1-AS1
MIR1284
EIF4E3
GPR27
PROK2
LINC00877
LINC00870
RYBP
$-\log_{10}(p\text{-value})$
Recombination rate (cM/Mb)
r^2

d

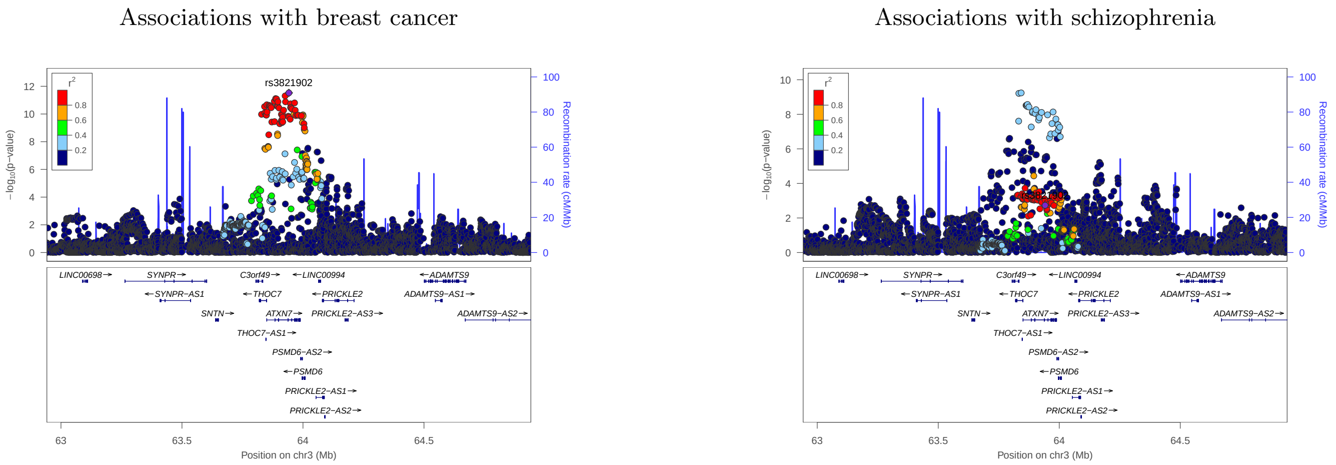
Associations with breast cancer
Associations with schizophrenia
rs3821902
r2
0.8
0.6
0.4
0.2
−log10(p−value)
Recombination rate (cM/Mb)
LINC00698
SYNPR
SYNPR−AS1
SNTN
C3orf49
THOC7
THOC7−AS1
ATXN7
LINC00994
PRICKLE2
PRICKLE2−AS3
PSMD6−AS2
PSMD6
PRICKLE2−AS1
PRICKLE2−AS2
ADAMTS9
ADAMTS9−AS1
ADAMTS9−AS2
Position on chr3 (Mb)

e

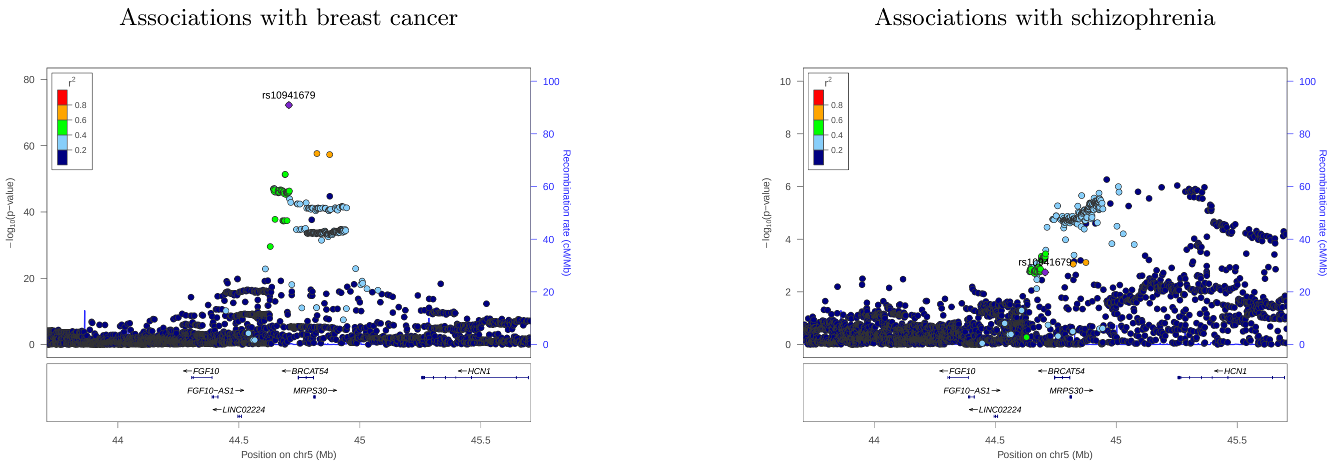
Associations with breast cancer
Associations with schizophrenia
rs10941679
r2
0.8
0.6
0.4
0.2
−log10(p−value)
Recombination rate (cM/Mb)
FGF10
FGF10−AS1
LINC02224
BRCAT54
MRPS30
HCN1
Position on chr5 (Mb)

f

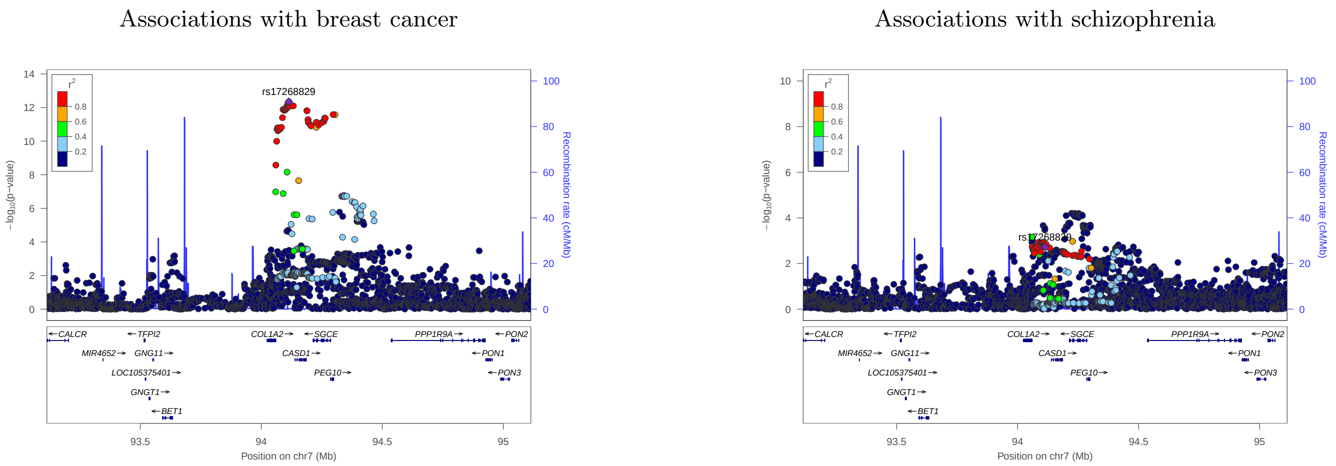
Associations with breast cancer
Associations with schizophrenia
rs17268829
r2
0.8
0.6
0.4
0.2
−log10(p−value)
Recombination rate (cM/Mb)
CALCR
MIR4652
TFPI2
GNG11
LOC105375401
GNGT1
BET1
COL1A2
CASD1
SGCE
PEG10
PPP1R9A
PON1
PON2
PON3
Position on chr7 (Mb)

g

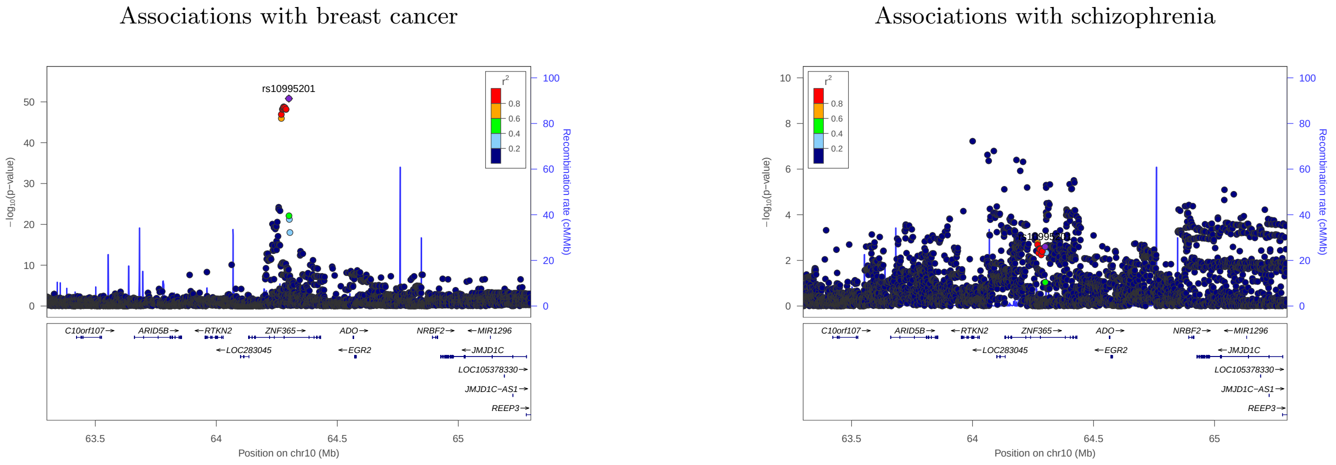
Associations with breast cancer
Associations with schizophrenia
rs10995201
−log10(p−value)
Recombination rate (cM/Mb)
r2
0.8
0.6
0.4
0.2
C10orf107
ARID5B
RTKN2
ZNF365
ADO
NRBF2
MIR1296
LOC283045
EGR2
JMJD1C
LOC105378330
JMJD1C−AS1
REEP3
Position on chr10 (Mb)

h

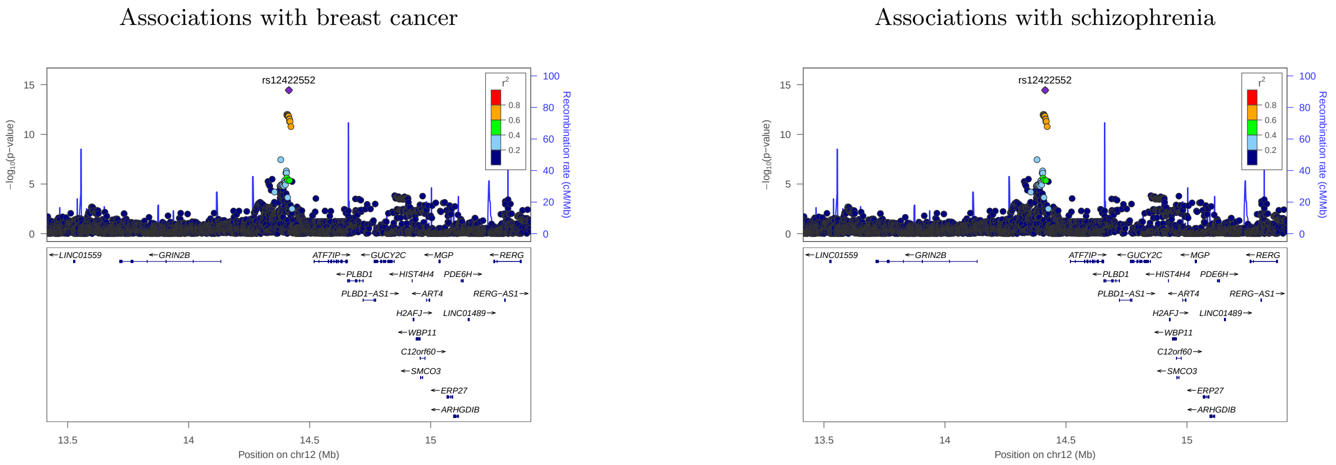
Associations with breast cancer
Associations with schizophrenia
rs12422552
−log10(p−value)
Recombination rate (cM/Mb)
r2
0.8
0.6
0.4
0.2
LINC01559
GRIN2B
ATF7IP
GUCY2C
MGP
RERG
PLBD1
HIST4H4
PDE6H
PLBD1−AS1
ART4
RERG−AS1
H2AFJ
LINC01489
WBP11
C12orf60
SMCO3
ERP27
ARHGDIB
Position on chr12 (Mb)

i

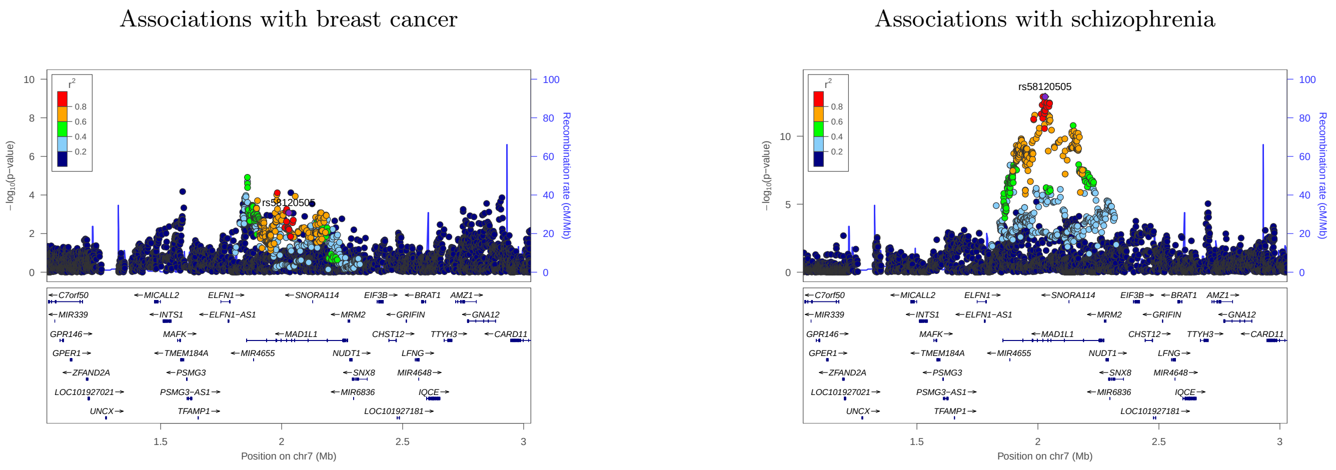
Associations with breast cancer
Associations with schizophrenia
rs58120505
−log10(p−value)
Recombination rate (cM/Mb)
r2
0.8
0.6
0.4
0.2
C7orf50
MICALL2
ELFN1
SNORA114
EIF3B
BRAT1
AMZ1
MIR339
INTS1
ELFN1−AS1
MRM2
GRIFIN
GNA12
GPR146
MAFK
MAD1L1
CHST12
TTYH3
CARD11
GPER1
TMEM184A
MIR4655
NUDT1
LFNG
ZFAND2A
PSMG3
SNX8
MIR4648
LOC101927021
PSMG3−AS1
MIR6836
IQCE
UNCX
TFAMP1
LOC101927181
Position on chr7 (Mb)

j

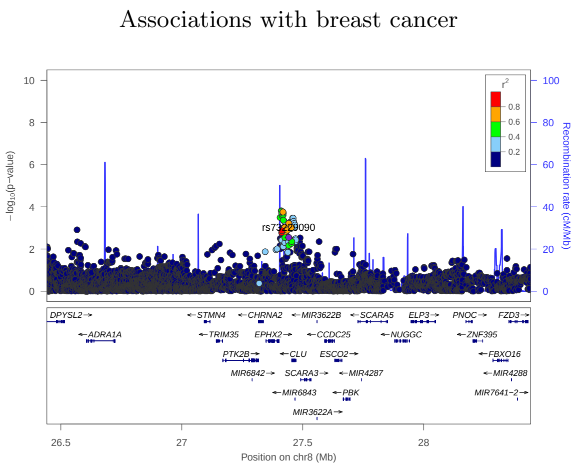
Associations with breast cancer
rs73229090
−log10(p−value)
Recombination rate (cM/Mb)
Position on chr8 (Mb)

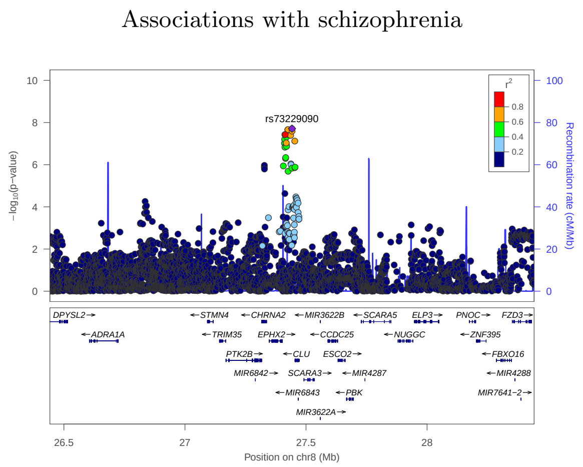
Associations with schizophrenia
rs73229090
−log10(p−value)
Recombination rate (cM/Mb)
Position on chr8 (Mb)

k

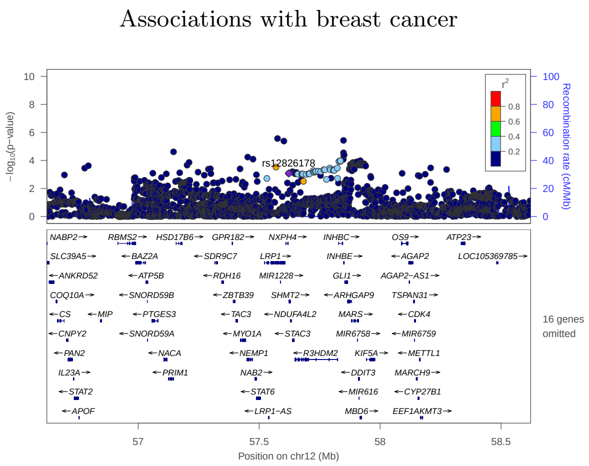
Associations with breast cancer
rs12826178
−log10(p−value)
Recombination rate (cM/Mb)
16 genes omitted
Position on chr12 (Mb)

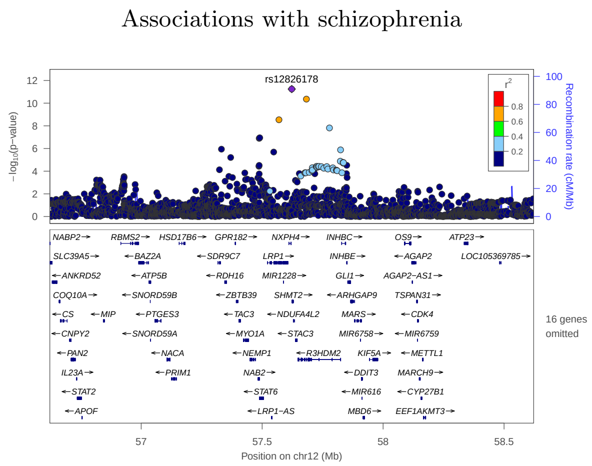
Associations with schizophrenia
rs12826178
−log10(p−value)
Recombination rate (cM/Mb)
16 genes omitted
Position on chr12 (Mb)

l

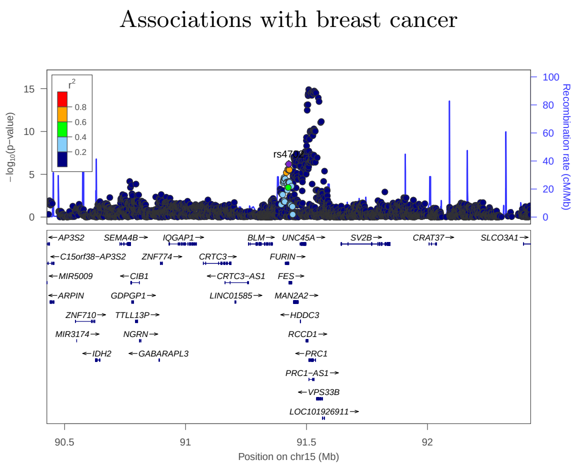
Associations with breast cancer
−log10(p−value)
Recombination rate (cM/Mb)
Position on chr15 (Mb)

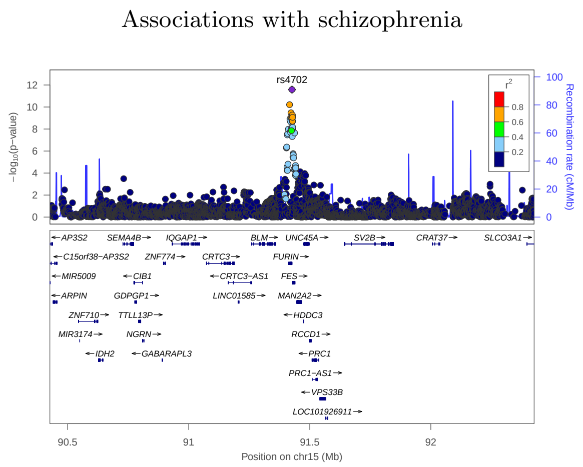
Associations with schizophrenia
rs4702
−log10(p−value)
Recombination rate (cM/Mb)
Position on chr15 (Mb)

m

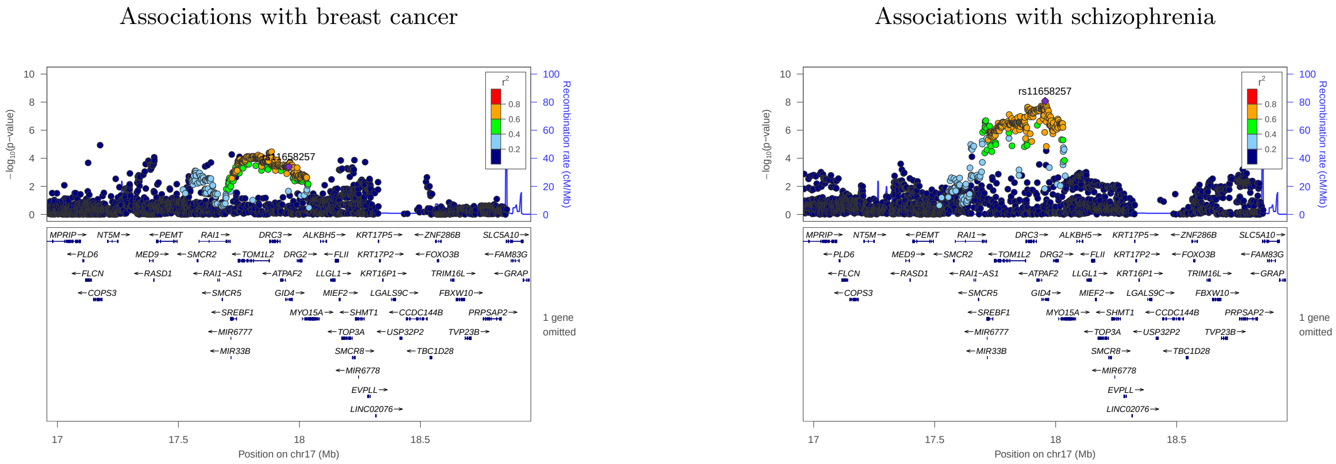

Supplementary Figure 3. Regional association plot of GWAS summary statistics from breast cancer and schizophrenia for loci anchored by lead SNPs for breast cancer and schizophrenia, respectively. a, Locus 2q33: rs3769821 (lead SNP for breast cancer). b, Locus 2q33: rs3769821 (lead SNP for breast cancer). c, Locus 3p13: rs6805189 (lead SNP for breast cancer). d, Locus 3p14: rs3821902 (lead SNP for breast cancer). e, Locus 5p12: rs10941679 (lead SNP for breast cancer). f, Locus 7q21: rs17268829 (lead SNP for breast cancer). g, Locus 10q21: rs10995201 (lead SNP for breast cancer). h, Locus 12p13: rs12422552 (lead SNP for breast cancer). i, Locus 7p22: rs58120505 (lead SNP for schizophrenia). j, Locus 8p21: rs73229090 (lead SNP for schizophrenia). k, Locus 12q13: rs12826178 (lead SNP for schizophrenia). l, Locus 15q26: rs4702 (lead SNP for schizophrenia). m, Locus 17p11: rs11658257 (lead SNP for schizophrenia). The gradient of color denotes the extent of LD (i.e., lighter color means lower LD) to the lead SNP. GWAS, genome-wide association study; LD, linkage disequilibrium; SNPs, single nucleotide polymorphisms.

a

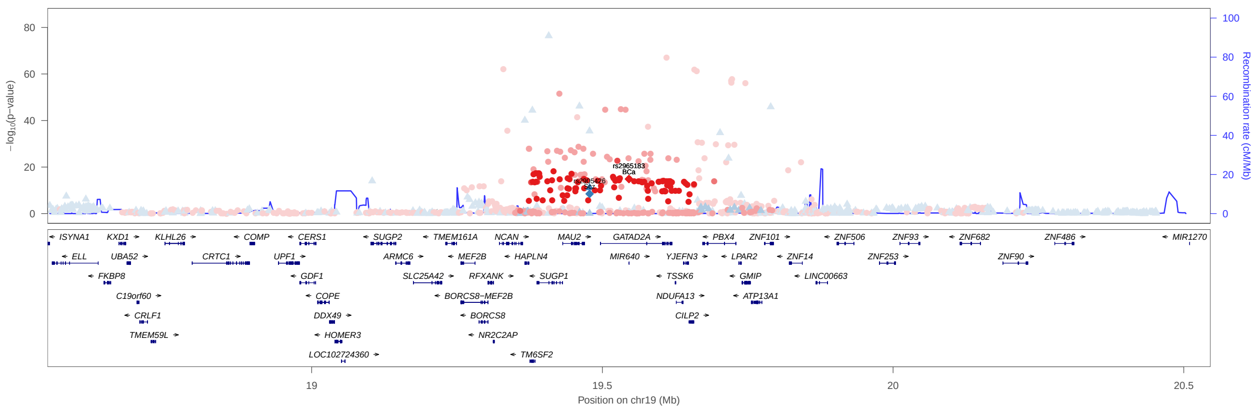

b

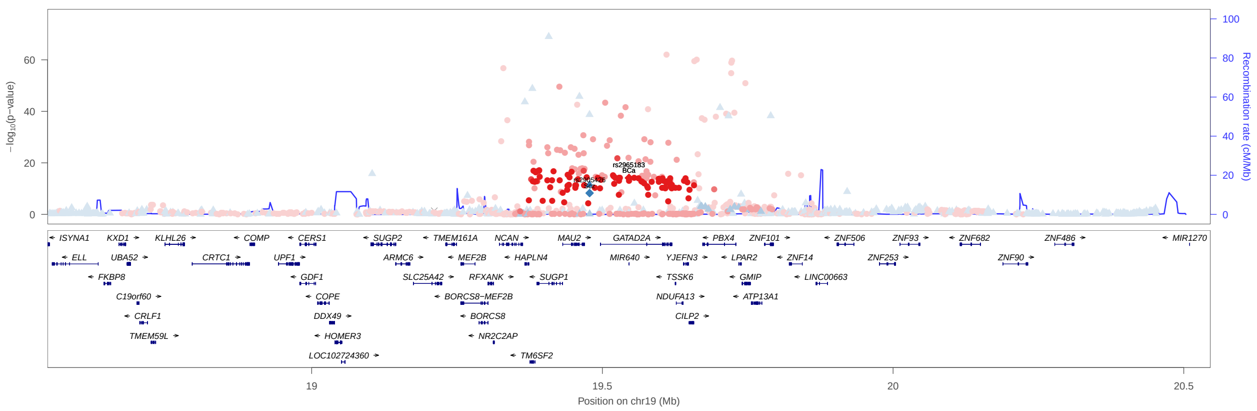

c

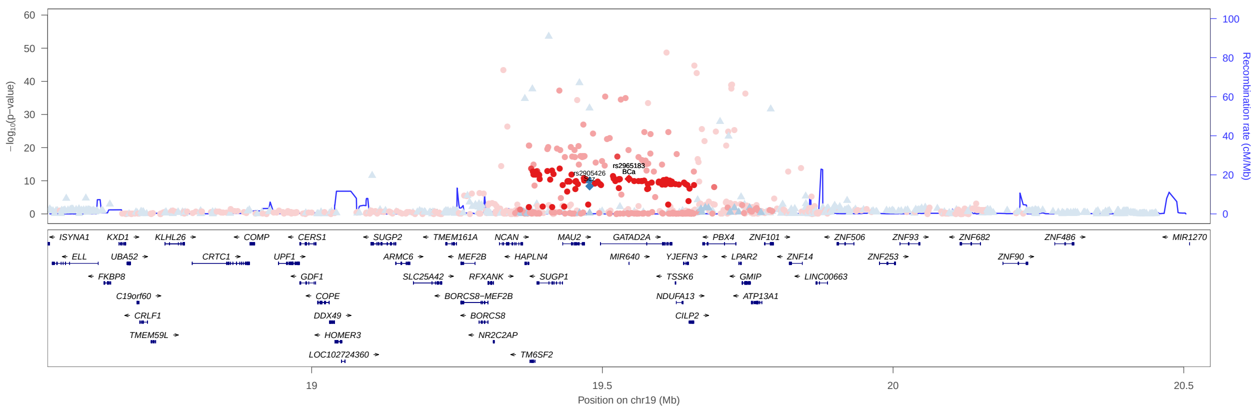

Supplementary Figure 4. Regional association plot of GWAS summary statistics from total cholesterol (TC), triglycerides (TG), and low-density lipoprotein (LDL) for the locus 19p13. a, Associations with TC. b, Associations with TG. c, Associations with LDL. This locus is anchored by lead SNPs rs2965183 (red) and rs2905426 (blue) for breast cancer and schizophrenia, respectively. SNPs are colored corresponding to which of the lead SNPs it is in highest LD with, and the gradient of color denotes the extent of LD (i.e., the lighter color means lower LD). GWAS, genome-wide association study; LD, linkage disequilibrium; SNPs, single nucleotide polymorphisms.

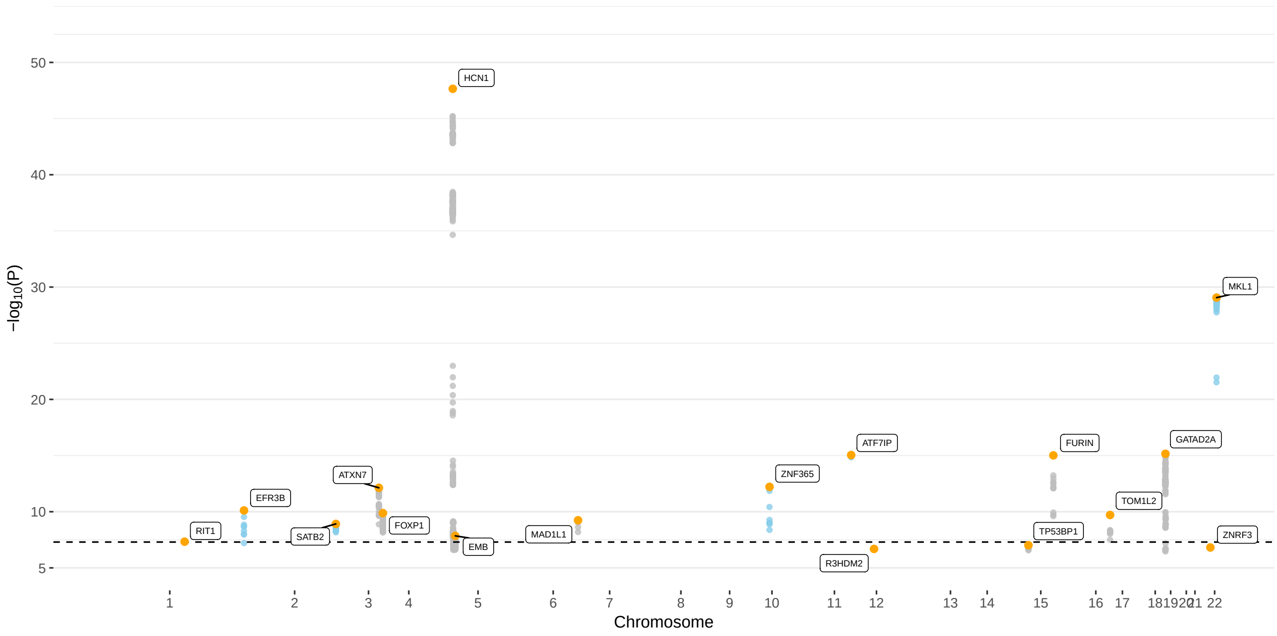

Supplementary Figure 5. Manhattan plot showing the result of cross-trait meta-analysis of GWASs on breast cancer and schizophrenia, highlighting the lead SNPs (orange), and the nearest genes (box). The analysis was restricted to 946 SNPs of P<5e-5 for both breast cancer and schizophrenia.

Supplementary Table 1. Bidirectional association between invasive breast cancer and early-onset schizophrenia among women of age at follow-up during 18-44 years.

	Women without breast cancer	Women with breast cancer				
	N (%)	N (%)	OR (95% CI)‡	*P* ‡	OR (95% CI)¶	*P* ‡
Association of schizophrenia with subsequent breast cancer*						
Number of women	258,450	8,615	-	-	-	-
Inpatient diagnosis of schizophrenia	900 (0.35	32 (0.37)	1.10 (0.77-1.56)	0.61	1.08 (0.76-1.55)	0.666
	N (%)	N (%)	HR (95% CI)‡	*P* ‡	HR (95% CI)¶	*P* ‡
Association of breast cancer with subsequent schizophrenia†						
Number of women	257,550	8,583	-	-	-	-
Inpatient diagnosis of schizophrenia	248 (0.10)	12 (0.14)	1.78 (0.98-3.21)	0.057	1.52 (0.69-3.38)	0.302

N, number; OR, odds ratio; HR, hazard ratio.

* Based on the nested case-control study. The estimates, i.e., OR derived from conditional logistic regression, should be interchangeably interpreted as the risk of breast cancer among patients with schizophrenia.

† Based on the matched cohort study. The estimates, i.e., HR derived from stratified Cox proportional hazards regression, are interpreted as the risk of schizophrenia among patients with breast cancer.

‡ Models were adjusted for educational level (primary school, high school, college and beyond, or unknown), and region of residence (southern, central, or northern Sweden). Birth year and age at reference were inherently controlled for due to matching.

¶ Models were additionally adjusted for parity (0, 1-2, or $\geq$3), pre-existing psychiatric disorder (yes or no; including substance use disorders), and obesity (yes or no) at matching.

Supplementary Table 2. Polygenic risk scores (PRS) for breast cancer and schizophrenia risk in women and men based on individual-level genotype data from Swedish Schizophrenia Study (S3) in Sweden.

PRS profile	Women (1983 cases + 3026 controls)			Men (2941 cases + 3181 controls)	
	OR (95% CI)	*P*		OR (95% CI)	*P*
$P_{bc} < 5\times10^{-8}$	0.97 (0.92-1.03)	0.33		1.00 (0.95-1.06)	0.88
$P_{bc} < 1\times10^{-6}$	0.99 (0.93-1.05)	0.67		0.99 (0.94-1.04)	0.73
$P_{bc} < 1\times10^{-4}$	1.02 (0.96-1.08)	0.55		0.99 (0.94-1.04)	0.70
$P_{bc} < 0.001$	1.02 (0.97-1.08)	0.45		1.01 (0.96-1.06)	0.75
$P_{bc} < 0.01$	1.07 (1.01-1.13)	0.03		1.03 (0.98-1.09)	0.24
$P_{bc} < 0.05$	1.08 (1.02-1.14)	0.01		1.04 (0.99-1.10)	0.10
$P_{bc} < 0.1$	1.08 (1.02-1.15)	0.008		1.05 (1.00-1.11)	0.06
$P_{bc} < 0.2$	1.09 (1.03-1.16)	0.004		1.07 (1.01-1.13)	0.01
$P_{bc} < 0.5$	1.09 (1.03-1.16)	0.003		1.07 (1.01-1.13)	0.02
$P_{bc} < 1$	1.09 (1.03-1.16)	0.005		1.07 (1.01-1.13)	0.02

OR, odds ratio; CI, confidence interval.

Supplementary Table 3. Known associations of lead SNPs rs2965183 and rs2905426 in locus 19p13.

Chromosome	Position	SNP	A1	A2	Effect (coefficient)	*P*	Phenotypes	N of participants	N of cases	N of controls	Ancestry	Study
19	19478022	rs2905426	G	T	0.04	8.401E-12	Breast cancer	228,951	122,977	105,974	European	PMID:25751625 [1]
19	19478022	rs2905426	G	T	-0.03	3.325E-09	Total cholesterol	80,250	N/A	N/A	European	PMID:24097068 [2]
19	19478022	rs2905426	G	T	0.07	4.067E-09	Schizophrenia	150,064	36,989	113,075	European	PMID:25056061 [3]
19	19478022	rs2905426	G	T	-0.04	4.323E-09	LDL	70,829	N/A	N/A	European	PMID:24097068 [2]
19	19478022	rs2905426	G	T	-0.03	5.424E-09	Triglycerides	74,374	N/A	N/A	European	PMID:24097068 [2]
19	19545696	rs2965183	A	G	-0.03	1.419E-15	Total cholesterol	180,262	N/A	N/A	European	PMID:24097068 [2]
19	19545696	rs2965183	A	G	-0.03	7.098E-15	Triglycerides	170,774	N/A	N/A	European	PMID:24097068 [2]
19	19545696	rs2965183	A	G	0.04	6.314E-12	Breast cancer	228,951	122,977	105,974	European	PMID:25751625 [1]
19	19545696	rs2965183	A	G	-0.03	2.839E-11	LDL	166,110	N/A	N/A	European	PMID:24097068 [2]
19	19545696	rs2965183	A	G	0.06	1.68E-08	Schizophrenia	150,064	36,989	113,075	European	PMID:25056061 [3]

SNP, single nucleotide polymorphism; A1, effect allele; A2, reference allele; N, number; N/A, not applicable.

Supplementary Table 4. Known associations in locus 19p13 and r-squares to rs2965183 and rs2905426.

Chr	Position	SNP	*P*	Mapped Genes	Phenotype	Study	r^2 to rs296518	r^2 to rs290542
Lipids								
19	19252779	rs190121281	5.00E-23	*TMEM161A-MEF2BNB-MEF2B*	lipid measurement, blood metabolite measurement	PMID:27005778 [4]	0.003	0.002
19	19329924	rs2228603	1.00E-35	*NCAN*	total cholesterol measurement, C-reactive protein measurement	PMID:27286809 [5]	0.107	0.11
19	19329924	rs2228603	7.00E-21	*NCAN*	low density lipoprotein cholesterol measurement, C-reactive protein measurement	PMID:27286809 [5]	0.107	0.11
19	19336608	rs2238675	1.00E-13	*NCAN*	total cholesterol measurement	PMID:26780889 [6]	0.04	0.042
19	19336608	rs2238675	2.00E-08	*NCAN*	low density lipoprotein cholesterol measurement	PMID:26780889 [6]	0.04	0.042
19	19366632	rs72999033	1.00E-16	*HAPLN4*	lipoprotein measurement, blood metabolite measurement	PMID:27005778 [4]	0.125	0.128
19	19370341	rs150641967	3.00E-11	*HAPLN4*	low density lipoprotein cholesterol measurement	PMID:28548082 [7]	0.143	0.147
19	19370341	rs150641967	8.00E-11	*HAPLN4*	total cholesterol measurement	PMID:28548082 [7]	0.143	0.147
19	19370341	rs150641967	1.00E-08	*HAPLN4*	very low density lipoprotein cholesterol measurement	PMID:28548082 [7]	0.143	0.147
19	19370341	rs150641967	2.00E-08	*HAPLN4*	triglyceride measurement	PMID:28548082 [7]	0.143	0.147
19	19379549	rs58542926	8.00E-29	*TM6SF2*	total cholesterol measurement	PMID:25961943 [8]	0.143	0.147
19	19379549	rs58542926	2.00E-25	*TM6SF2*	triglyceride measurement	PMID:25961943 [8]	0.143	0.147
19	19407718	rs10401969	4.00E-77	*SUGP1*	total cholesterol measurement	PMID:24097068 [2]	0.149	0.153
19	19407718	rs10401969	5.00E-71	*SUGP1*	triglyceride measurement	PMID:28334899 [9]	0.149	0.153
19	19407718	rs10401969	3.00E-54	*SUGP1*	low density lipoprotein cholesterol measurement	PMID:24097068 [2]	0.149	0.153
19	19407718	rs10401969	2.00E-32	*SUGP1*	triglyceride measurement, C-reactive protein measurement	PMID:27286809 [5]	0.149	0.153
19	19407718	rs10401969	2.00E-11	*SUGP1*	total cholesterol measurement, diastolic blood pressure, triglyceride measurement, systolic blood pressure, hematocrit, ventricular rate measurement, glucose measurement, body mass index, high density lipoprotein cholesterol measurement	PMID:27790247 [10]	0.149	0.153
19	19407718	rs10401969	4.00E-11	*SUGP1*	total cholesterol measurement, hematocrit, stroke, ventricular rate measurement, coronary heart disease, body mass index, atrial fibrillation, high density lipoprotein cholesterol measurement, cancer, diastolic blood pressure, triglyceride measurement, systolic blood pressure, heart failure, diabetes mellitus, glucose measurement, mortality	PMID:27790247 [10]	0.149	0.153
19	19455750	rs8102280	3.00E-18	*MAU2*	triglyceride measurement	PMID:26780889 [6]	0.008	0.008
19	19460541	rs73001065	3.00E-23	*MAU2*	low density lipoprotein cholesterol measurement	PMID:25961943 [8]	0.134	0.137
19	19658472	rs16996148	3.00E-09	*CILP2 - PBX4*	low density lipoprotein cholesterol measurement	PMID:18193043 [11]	0.159	0.156
19	19658472	rs16996148	3.00E-09	*CILP2 - PBX4*	triglyceride measurement	PMID:18193043 [11]	0.159	0.156
19	19662220	rs17216525	4.00E-11	*CILP2 - PBX4*	triglyceride measurement	PMID:19060906 [12]	0.154	0.152
19	19756074	rs3841260	2.00E-08	*ATP13A1*	LDL cholesterol change measurement, response to fenofibrate	PMID:27002377 [13]	0.125	0.128
19	19789528	rs2304130	2.00E-15	*ZNF101*	total cholesterol measurement	PMID:19060911 [14]	0.129	0.133
19	19789528	rs2304130	6.00E-09	*ZNF101*	sphingolipid measurement	PMID:22359512 [15]	0.129	0.133
19	19789528	rs2304130	4.00E-08	*ZNF101*	triglyceride measurement	PMID:20864672 [16]	0.129	0.133
Blood count								
19	19467937	rs2285627	1.00E-09	*MAU2*	basophil count	PMID:27863252 [17]	0.813	0.836
19	19657632	rs7245983	2.00E-15	*CILP2 - PBX4*	red blood cell distribution width	PMID:27863252 [17]	0.871	0.886
19	19702384	rs17217098	9.00E-10	*PBX4*	lymphocyte percentage of leukocytes	PMID:27863252 [17]	0.13	0.133
19	19756074	rs3841260	4.00E-31	*ATP13A1*	platelet crit	PMID:27863252 [17]	0.125	0.128
19	19756074	rs3841260	6.00E-21	*ATP13A1*	platelet count	PMID:27863252 [17]	0.125	0.128
19	19670688	rs7249692	2.00E-09	*CILP2 - PBX4*	monocyte count	PMID:27863252 [17]	0.548	0.558
19	19789425	rs71172513	3.00E-11	*ZNF101*	basophil percentage of granulocytes	PMID:27863252 [17]	0.165	0.171
Psychobehavioral profiles								
19	19299079	rs8100480	3.00E-08	*MEF2BNB-MEF2B, MEF2BNB*	obsessive-compulsive symptom measurement	PMID:26859814 [18]	0.029	0.031
19	19361735	rs1064395	2.00E-09	*NCAN*	bipolar disorder	PMID:21353194 [19]	0.357	0.388
19	19473445	rs2905424	3.00E-09	*MAU2 - GATAD2A*	schizophrenia	PMID:23974872 [20]	0.904	0.929
19	19478022	rs2905426	4.00E-10	*MAU2 - GATAD2A*	schizophrenia	PMID:25056061 [3]	0.93	1
19	19513570	rs111901094	3.00E-09	*GATAD2A*	physical activity measurement	PMID:29899525 [21]	0.249	0.249
19	19654117	rs7252453	4.00E-14	*CILP2*	mathematical ability	PMID:30038396 [22]	0.404	0.415
19	19663850	rs892023	7.00E-11	*CILP2 - PBX4*	self-reported educational attainment	PMID:30038396 [22]	0.018	0.024
Others								
19	19260686	rs75746498	9.00E-09	*MEF2BNB-MEF2B, MEF2B*	diastolic blood pressure	PMID:29403010 [23]	0.008	0.008
19	19263938	rs58190593	1.00E-09	*MEF2BNB-MEF2B, MEF2B*	mean arterial pressure	PMID:29403010 [23]	0.074	0.077
19	19407718	rs10401969	8.00E-10	*SUGP1*	alcoholic liver cirrhosis	PMID:26482880 [24]	0.149	0.153
19	19407718	rs10401969	7.00E-09	*SUGP1*	type II diabetes mellitus	PMID:22885922 [25]	0.149	0.153
19	19545099	rs4808199	2.00E-08	*GATAD2A*	non-alcoholic fatty liver disease	PMID:29385134 [26]	0.356	0.358
19	19545696	rs2965183	6.00E-12	*GATAD2A*	breast cancer	PMID:29059683 [27]	1	0.93
19	19579557	rs4808962	1.00E-09	*GATAD2A*	age at onset, Myopia, refractive error measurement	PMID:29808027 [28]	0.403	0.414
19	19591066	rs10401193	9.00E-14	*GATAD2A*	body height	PMID:25282103 [29]	0.356	0.358
19	19610596	rs3794991	9.00E-13	*GATAD2A*	type II diabetes mellitus	PMID:28869590 [30]	0.165	0.163
19	19789528	rs2304130	2.00E-08	*ZNF101*	diastolic blood pressure	PMID:27618447 [31]	0.129	0.133

Chr, chromosome; SNP, single nucleotide polymorphism.

Supplementary Table 5. Expression Quantitative Trait Loci (eQTL) analysis results from the GTEx Portal for the lead SNPs rs2965183 and rs2905426 in locus 19p13.

SNP	Chromosome	Position	A1	Gene	Effect (coefficient)	Empirical *P*	Corrected *P*	Tissue
rs2905426	19	19478022	G	*TM6SF2*	0.53	1.56E-08	9.04E-06	Brain Cerebellum
rs2905426	19	19478022	G	*HAPLN4*	0.18	1.67E-05	1.90E-04	Brain Cerebellum
rs2905426	19	19478022	G	*GATAD2A*	0.19	1.61E-15	3.34E-15	Whole Blood
rs2905426	19	19478022	G	*LPAR2*	-0.13	1.86E-07	1.83E-18	Whole Blood
rs2905426	19	19478022	G	*TSSK6*	0.20	2.49E-07	4.15E-05	Whole Blood
rs2905426	19	19478022	G	*MAU2*	-0.08	2.01E-05	2.19E-04	Whole Blood
rs2965183	19	19545696	A	*TM6SF2*	0.42	2.14E-05	7.26E-03	Brain Cerebellar Hemisphere
rs2965183	19	19545696	A	*TM6SF2*	0.51	7.61E-08	9.04E-06	Brain Cerebellum
rs2965183	19	19545696	A	*HAPLN4*	0.27	1.72E-05	1.42E-03	Breast Mammary Tissue
rs2965183	19	19545696	A	*GATAD2A*	0.21	2.64E-19	3.34E-15	Whole Blood
rs2965183	19	19545696	A	*TSSK6*	0.20	5.38E-08	4.15E-05	Whole Blood
rs2965183	19	19545696	A	*LPAR2*	-0.13	6.91E-08	1.83E-18	Whole Blood
rs2965183	19	19545696	A	*MAU2*	-0.08	3.80E-05	2.19E-04	Whole Blood

SNP, single nucleotide polymorphism; A1, effect allele.

Supplementary Table 6. Swedish version of International Classification of Diseases (ICD) codes for breast cancer, psychiatric disorders and obesity.

Cancer Register	ICD-7 (1958-2009)		
Breast Cancer	170		
Patient Register	ICD-8 (1981-1986)	ICD-9 (1987-1996)	ICD-10 (1997-2010)
Schizophrenia	295	295	F20
Other psychiatric disorders	291, 296-319	291, 292, 296-319	F10-F19, F21-F99
Obesity	277.99	278A, 278B	E65, E66

Supplementary References

1. Michailidou, K. *et al.* Genome-wide association analysis of more than 120,000 individuals identifies 15 new susceptibility loci for breast cancer. *Nat Genet* **47**, 373-80 (2015).
2. Willer, C.J. *et al.* Discovery and refinement of loci associated with lipid levels. *Nat Genet* **45**, 1274-1283 (2013).
3. Schizophrenia Working Group of the Psychiatric Genomics, C. Biological insights from 108 schizophrenia-associated genetic loci. *Nature* **511**, 421-7 (2014).
4. Kettunen, J. *et al.* Genome-wide study for circulating metabolites identifies 62 loci and reveals novel systemic effects of LPA. *Nat Commun* **7**, 11122 (2016).
5. Ligthart, S. *et al.* Bivariate genome-wide association study identifies novel pleiotropic loci for lipids and inflammation. *BMC Genomics* **17**, 443 (2016).
6. Below, J.E. *et al.* Meta-analysis of lipid-traits in Hispanics identifies novel loci, population-specific effects, and tissue-specific enrichment of eQTLs. *Sci Rep* **6**, 19429 (2016).
7. Southam, L. *et al.* Whole genome sequencing and imputation in isolated populations identify genetic associations with medically-relevant complex traits. *Nat Commun* **8**, 15606 (2017).
8. Surakka, I. *et al.* The impact of low-frequency and rare variants on lipid levels. *Nat Genet* **47**, 589-97 (2015).
9. Spracklen, C.N. *et al.* Association analyses of East Asian individuals and trans-ancestry analyses with European individuals reveal new loci associated with cholesterol and triglyceride levels. *Hum Mol Genet* **26**, 1770-1784 (2017).
10. He, L. *et al.* Pleiotropic Meta-Analyses of Longitudinal Studies Discover Novel Genetic Variants Associated with Age-Related Diseases. *Front Genet* **7**, 179 (2016).
11. Willer, C.J. *et al.* Newly identified loci that influence lipid concentrations and risk of coronary artery disease. *Nat Genet* **40**, 161-9 (2008).
12. Kathiresan, S. *et al.* Common variants at 30 loci contribute to polygenic dyslipidemia. *Nat Genet* **41**, 56-65 (2009).
13. Irvin, M.R. *et al.* A genome-wide study of lipid response to fenofibrate in Caucasians: a combined analysis of the GOLDN and ACCORD studies. *Pharmacogenet Genomics* **26**, 324-33 (2016).
14. Aulchenko, Y.S. *et al.* Loci influencing lipid levels and coronary heart disease risk in 16 European population cohorts. *Nat Genet* **41**, 47-55 (2009).
15. Demirkan, A. *et al.* Genome-wide association study identifies novel loci associated with circulating phospho- and sphingolipid concentrations. *PLoS Genet* **8**, e1002490 (2012).
16. Waterworth, D.M. *et al.* Genetic variants influencing circulating lipid levels and risk of coronary artery disease. *Arterioscler Thromb Vasc Biol* **30**, 2264-76 (2010).
17. Astle, W.J. *et al.* The Allelic Landscape of Human Blood Cell Trait Variation and Links to Common Complex Disease. *Cell* **167**, 1415-1429.e19 (2016).
18. den Braber, A. *et al.* Obsessive-compulsive symptoms in a large population-based twin-family sample are predicted by clinically based polygenic scores and by genome-wide SNPs. *Transl Psychiatry* **6**, e731 (2016).
19. Cichon, S. *et al.* Genome-wide association study identifies genetic variation in neurocan as a susceptibility factor for bipolar disorder. *Am J Hum Genet* **88**, 372-81 (2011).

20. Ripke, S. *et al.* Genome-wide association analysis identifies 13 new risk loci for schizophrenia. *Nat Genet* **45**, 1150-9 (2013).
21. Klimentidis, Y.C. *et al.* Genome-wide association study of habitual physical activity in over 377,000 UK Biobank participants identifies multiple variants including CADM2 and APOE. *Int J Obes (Lond)* **42**, 1161-1176 (2018).
22. Lee, J.J. *et al.* Gene discovery and polygenic prediction from a genome-wide association study of educational attainment in 1.1 million individuals. *Nat Genet* **50**, 1112-1121 (2018).
23. Kanai, M. *et al.* Genetic analysis of quantitative traits in the Japanese population links cell types to complex human diseases. *Nat Genet* **50**, 390-400 (2018).
24. Buch, S. *et al.* A genome-wide association study confirms PNPLA3 and identifies TM6SF2 and MBOAT7 as risk loci for alcohol-related cirrhosis. *Nat Genet* **47**, 1443-8 (2015).
25. Morris, A.P. *et al.* Large-scale association analysis provides insights into the genetic architecture and pathophysiology of type 2 diabetes. *Nat Genet* **44**, 981-90 (2012).
26. Kawaguchi, T. *et al.* Risk estimation model for nonalcoholic fatty liver disease in the Japanese using multiple genetic markers. *PLoS One* **13**, e0185490 (2018).
27. Michailidou, K. *et al.* Association analysis identifies 65 new breast cancer risk loci. *Nature* **551**, 92-94 (2017).
28. Tedja, M.S. *et al.* Genome-wide association meta-analysis highlights light-induced signaling as a driver for refractive error. *Nat Genet* **50**, 834-848 (2018).
29. Wood, A.R. *et al.* Defining the role of common variation in the genomic and biological architecture of adult human height. *Nat Genet* **46**, 1173-86 (2014).
30. Zhao, W. *et al.* Identification of new susceptibility loci for type 2 diabetes and shared etiological pathways with coronary heart disease. *Nat Genet* **49**, 1450-1457 (2017).
31. Surendran, P. *et al.* Trans-ancestry meta-analyses identify rare and common variants associated with blood pressure and hypertension. *Nat Genet* **48**, 1151-1161 (2016).
