# Supplementary material for: A shared genetic contribution to breast cancer and schizophrenia
Source: Nat Commun. 2020 Sep 15;11:4637. doi: 10.1038/s41467-020-18492-8 (PMC7492262; doi:10.1038/s41467-020-18492-8)
Supplement: Supplementary file 3 — Descriptions of Additional Supplementary Files [file 41467_2020_18492_MOESM3_ESM.pdf]

Descriptions of Additional Supplementary Files

Supplementary Data 1.

Description: Pathway analysis results for pathways with corrected $P<0.05$. 84 genes mapped according to position or eQTL in GTEx (breast, brain, and blood samples) were included for pathway analysis.
