# Supplementary material for: A shared genetic contribution to breast cancer and schizophrenia
Source: Nat Commun. 2020 Sep 15;11:4637. doi: 10.1038/s41467-020-18492-8 (PMC7492262; doi:10.1038/s41467-020-18492-8)
Supplement: Supplementary file 5 — Reporting Summary [file 41467_2020_18492_MOESM5_ESM.pdf]

nature research

Corresponding author(s): Donghao Lu, Jie Song

Last updated by author(s): Aug 12, 2020

Reporting Summary

Nature Research wishes to improve the reproducibility of the work that we publish. This form provides structure for consistency and transparency in reporting. For further information on Nature Research policies, see our Editorial Policies and the Editorial Policy Checklist.

Statistics

For all statistical analyses, confirm that the following items are present in the figure legend, table legend, main text, or Methods section.

n/a	Confirmed	
☐	☒	The exact sample size (*n*) for each experimental group/condition, given as a discrete number and unit of measurement
☒	☐	A statement on whether measurements were taken from distinct samples or whether the same sample was measured repeatedly
☐	☒	The statistical test(s) used AND whether they are one- or two-sided<br>*Only common tests should be described solely by name; describe more complex techniques in the Methods section.*
☐	☒	A description of all covariates tested
☐	☒	A description of any assumptions or corrections, such as tests of normality and adjustment for multiple comparisons
☐	☒	A full description of the statistical parameters including central tendency (e.g. means) or other basic estimates (e.g. regression coefficient) AND variation (e.g. standard deviation) or associated estimates of uncertainty (e.g. confidence intervals)
☐	☒	For null hypothesis testing, the test statistic (e.g. *F*, *t*, *r*) with confidence intervals, effect sizes, degrees of freedom and *P* value noted<br>*Give P values as exact values whenever suitable.*
☒	☐	For Bayesian analysis, information on the choice of priors and Markov chain Monte Carlo settings
☒	☐	For hierarchical and complex designs, identification of the appropriate level for tests and full reporting of outcomes
☐	☒	Estimates of effect sizes (e.g. Cohen's *d*, Pearson's *r*), indicating how they were calculated

Our web collection on statistics for biologists contains articles on many of the points above.

Software and code

Policy information about availability of computer code

Data collection	Not applicable
Data analysis	Data analyses were conducted in STATA v14.2, PRSice v1.25, PLINK v1.9, LDSC v1.0.1, R v3.4.3, and LocusZoom v1.4. All code used for data preparation and analysis are deposited on GitHub (https://github.com/donghaolu/bcascz).

For manuscripts utilizing custom algorithms or software that are central to the research but not yet described in published literature, software must be made available to editors and reviewers. We strongly encourage code deposition in a community repository (e.g. GitHub). See the Nature Research guidelines for submitting code & software for further information.

Data

Policy information about availability of data

All manuscripts must include a data availability statement. This statement should provide the following information, where applicable:

- Accession codes, unique identifiers, or web links for publicly available datasets
- A list of figures that have associated raw data
- A description of any restrictions on data availability

The epidemiological analysis used Swedish registers are available from the Swedish National Board of Health and Welfare (https://www.socialstyrelsen.se/en/statistics-and-data/registers/) and the Statistics Sweden (https://www.statistikdatabasen.scb.se/pxweb/en/ssd/). According to Swedish law, the authors are not able to make the dataset publicly available. Data are however available for any researchers (including international researchers) through formal applications to the aforementioned authorities. The primary genetic analysis used publicly available GWAS summary statistics from the Breast Cancer Association Consortium (available at http://bcac.ccge.medschl.cam.ac.uk/bcacdata/icogs-complete-summary-results/) and Psychiatric Genomics Consortium (available at https://www.med.unc.edu/pgc/download-results/). We also used reported genetic associations from PhenoScanner database (http://www.phenoscanner.medschl.cam.ac.uk/) and GWAS Catalog (https://www.ebi.ac.uk/gwas/home).

Field-specific reporting

Please select the one below that is the best fit for your research. If you are not sure, read the appropriate sections before making your selection.

- [x] Life sciences
- [ ] Behavioural & social sciences
- [ ] Ecological, evolutionary & environmental sciences

For a reference copy of the document with all sections, see nature.com/documents/nr-reporting-summary-flat.pdf

Life sciences study design

All studies must disclose on these points even when the disclosure is negative.

Sample size

In the epidemiological analysis, we included all women with invasive breast cancer (N=94,626) diagnosed in Sweden during 1990-2009 and matched cancer-free individuals randomly selected from the population (N=2,838,765). The nationwide approach gives us the maximal sample size we could have in this research setting.

In the genetic analysis, we also used the largest GWASs of breast cancer (122,977 cases and 105,974 controls) and schizophrenia (36,989 cases and 113,075) when the study was conducted.

Data exclusions

In the epidemiological analysis, since our study period was from 1990 onward, we excluded 39,990 women diagnosed with breast cancer, 81,692 diagnosed with other malignancy, and 48,320 with emigration before January 1, 1990, or age 18, whichever came later, and 982 with erroneous records. The exclusion criteria have been established in our previous study with a similar design (PMID 27124325).

In the genetic analysis, we restricted to the GWASs of European -ancestry individuals.

Replication

The PRS analysis using the Swedish Schizophrenia Study replicated our findings using the GWAS summary statistics. Given the nature of observational study, one independent replication is adequate.

Randomization

Randomization is not applicable as this is an observational study.

Blinding

Blinding is not applicable as this is an observational study.

Reporting for specific materials, systems and methods

We require information from authors about some types of materials, experimental systems and methods used in many studies. Here, indicate whether each material, system or method listed is relevant to your study. If you are not sure if a list item applies to your research, read the appropriate section before selecting a response.

Materials & experimental systems

n/a	Involved in the study	
x		Antibodies
x		Eukaryotic cell lines
x		Palaeontology and archaeology
x		Animals and other organisms
	x	Human research participants
x		Clinical data
x		Dual use research of concern

Methods

n/a	Involved in the study	
x		ChIP-seq
x		Flow cytometry
x		MRI-based neuroimaging

Human research participants

Policy information about studies involving human research participants

Population characteristics	Using nationwide population and health registers in Sweden, we identified all women with invasive breast cancer (N=94,626) diagnosed in Sweden during 1990-2009 (mean age at diagnosis, 63.6+/-14.1 years). For each cancer case, at the time of cancer diagnosis, we randomly selected 30 cancer-free women as controls (N=2,838,765) who were individually matched on birth year.
Recruitment	Based on the Swedish Population and Housing Census in 1990, we identified all women born and living in Sweden in 1990 (N=3,937,114). Using the unique national identification numbers, we followed all women through cross-linkages to the Swedish Cancer Register and Patient Register to identify occurring cases of breast cancer and schizophrenia, respectively. The nationwide coverage and prospective follow-up minimize common biases, e.g., selection and surveillance bias, in observational studies.
Ethics oversight	The register-based study was approved by the Central Ethical Review Board in Stockholm, Sweden (Dnr 12-2013). The S3 were approved by the Regional Ethical Review Board in Stockholm, Sweden (Dnr 04/449/4), and informed consent was obtained from every participant. Data from international consortia (summary statistics) are publicly available.

Note that full information on the approval of the study protocol must also be provided in the manuscript.
